# Determinants of footwear difficulties in people with plantar heel pain

**DOI:** 10.1186/s13047-015-0102-9

**Published:** 2015-08-18

**Authors:** Justin Sullivan, Evangelos Pappas, Roger Adams, Jack Crosbie, Joshua Burns

**Affiliations:** Discipline of Physiotherapy, Faculty of Health Sciences, The University of Sydney, Sydney, NSW Australia; School of Science and Health, The University of Western Sydney, Sydney, NSW Australia; Paediatric Gait Analysis Service of New South Wales, Sydney Children’s Hospitals Network (Randwick and Westmead), Sydney, NSW Australia; Arthritis and Musculoskeletal Research Group, Faculty of Health Sciences, The University of Sydney, Sydney, NSW Australia; Faculty of Health Sciences, The University of Sydney, P.O Box 170, Lidcombe, Sydney, NSW 1825 Australia

## Abstract

**Background:**

Plantar heel pain is a common foot disorder aggravated by weight-bearing activity. Despite considerable focus on therapeutic interventions such as orthoses, there has been limited investigation of footwear-related issues in people with plantar heel pain. The aim of this study was to investigate whether people with plantar heel pain experience footwear-related difficulties compared to asymptomatic individuals, as well as identifying factors associated with footwear comfort, fit and choice.

**Methods:**

The footwear domain of the Foot Health Status Questionnaire (FHSQ) was assessed in 192 people with plantar heel pain and 69 asymptomatic controls. The plantar heel pain group was also assessed on a variety of measures including: foot posture, foot strength and flexibility, pedobarography and pain level. A univariate analysis of covariance, with age as the covariate, was used to compare the heel pain and control groups on the FHSQ footwear domain score. A multiple regression model was then constructed to investigate factors associated with footwear scores among participants with plantar heel pain.

**Results:**

When compared to asymptomatic participants, people with plantar heel pain reported lower FHSQ footwear domain scores (mean difference −24.4; p < 0.001; 95 % CI: −32.0 to −17.0). In the participants with heel pain, footwear scores were associated with maximum force beneath the postero-lateral heel during barefoot walking, toe flexor strength and gender.

**Conclusions:**

People with plantar heel pain experience difficulty with footwear comfort, fit and choice. Reduced heel loading during barefoot walking, toe flexor weakness and female gender are all independently associated with reports of footwear difficulties in people with heel pain. Increased focus, in both clinical and research settings, is needed to address footwear-related issues in people with plantar heel pain.

## Background

Plantar heel pain is a common musculoskeletal condition that negatively impacts on both work and leisure activities [1]. It is the most common foot condition treated by physical therapists [2] and accounted for approximately one million physician consultations per year in the United States of America between 1995 and 2000 [3]. At present, there is limited evidence to support the numerous intervention strategies for plantar heel pain [4] and 11-18 % of people continue to report symptoms beyond 1 year following conservative management [5, 6]. Plantar heel pain is aggravated by weight-bearing activities [7], and although evidence supports mechanical treatment interventions such as orthoses [8–10] and taping [11, 12], little attention has focused on the role of footwear in plantar heel pain. One study, involving 80 people with chronic plantar heel pain, reported greater difficulties with footwear comfort, fit and choice compared to matched controls [13]. These data provide some evidence that footwear-related problems exist in people with heel pain; however, the biomechanical and musculoskeletal factors associated with footwear difficulties are unknown.

Difficulties relating to footwear can have adverse effects on individuals. Limitations in footwear choices can negatively impact self-image and limit social activities, as has been demonstrated in women with rheumatoid arthritis [14]. Furthermore, the mechanical nature of plantar heel pain [15] may implicate footwear as either a potential causative factor or as a therapeutic option. Although unsuitable or worn footwear, as well as changing footwear, are often suggested as potential causes of heel pain [16, 17], insufficient evidence exists regarding these aspects. Defective footwear has also been proposed as a cause of heel pain, but evidence for this factor is at case-study level only [18, 19]. Supportive shoes [16] and the avoidance of flat-soles [20] have been suggested as treatment options, although the effectiveness of these strategies has not been investigated. One study comparing the use of two different types of running shoe in the management of plantar heel pain reported potential benefits from the more flexible soled shoe [21]. Shoe modification has also been proposed as an intervention strategy for plantar heel pain [9, 22], with some benefit derived from rocker-sole footwear [23].

Despite promising investigations of footwear modification, limited evidence exists regarding the impact of heel pain on footwear comfort, fit and choice. The aim of this study was to investigate whether people with heel pain experience difficulties associated with footwear comfort, fit and choice, compared to healthy, asymptomatic people. In addition, we assessed the extent to which pain, anthropometric, biomechanical and musculoskeletal measures predict footwear-related difficulties in people with heel pain.

## Methods

202 people with plantar heel pain and 70 healthy controls volunteered to participate in this study. Complete data sets from 192 participants with heel pain and 69 controls were available for use in the analysis. A larger sample of heel pain participants was recruited in order to maximize power in the multiple regression analysis that would incorporate data from this group. Using the equation N ≥ 50 + 8 m (where N is the sample size and m is the number of independent variables), a sample size of 192 would enable up to 17 of the measures taken to be used as independent variables in the regression analysis [24]. People were recruited from the general community using local media advertisements, as well as notices in university, medical and physiotherapy premises. In accordance with current clinical guidelines [25], symptomatic participants were included if they were tender on palpation of the medial calcaneal tuberosity and exhibited one of the following complaints: pain on the first step in the morning or after prolonged sitting; pain on prolonged standing or walking [3, 25]; or pain when running [25]. Asymptomatic participants were required to have no past or present history of plantar heel pain. Participants were excluded from either group if they had undergone foot surgery, or had any of the following conditions: systemic arthritis, neurological conditions, lumbar radiculopathy, neurological or vascular compromise of the foot related to diabetes, peripheral neuropathies or any co-existing painful musculoskeletal condition of the lower limb.

Each participant attended data collection on a single occasion. In accordance with approval from the University of Sydney Human Research Ethics Committee, written consent was obtained and all participants were screened for the presence of plantar heel pain.

## Footwear comfort, fit and choice

Footwear comfort, fit and choice was assessed using the Foot Health Status Questionnaire (FHSQ) footwear domain, an instrument with high content, criterion, and construct validity, as well as test-retest reliability [26]. The footwear domain of the FHSQ consists of three footwear-related questions answered on a five-point Likert scale. The questions enquire regarding; (i) the ease of finding shoes that do not hurt; (ii) the ease of finding shoes that fit well; and (iii) any limitation in the number of different shoes able to be worn. Specific questionnaire software converts the responses of each question into a total score between 0 and 100 (FHSQ version 1.03). The minimum score represents very poor footwear comfort, fit and choice, with the maximum score representing no problem obtaining suitable footwear [26].

### Physical measures

#### Foot posture

The standing foot posture of each participant was measured using the Foot Posture Index (FPI), a reliable tool which scores an individual foot on a scale ranging from more supinated to more pronated in the weight-bearing position [27]. The FPI is a 25 point scale (from −12 to 12), using 5 observational and 1 palpation measure (each scored between −2 to 2) of the alignment of different segments of the foot and ankle (rearfoot, midfoot, and forefoot). A score of zero is given to neutral alignment; negative scores are given to more supinated alignment, and positive scores given to more pronated alignment. The average FPI score in the healthy population is 4, indicating that a slightly pronated foot alignment in standing is normal [28].

#### Muscle strength

The strength of the ankle dorsiflexors, invertors, evertors, and toe flexors was assessed using hand-held dynamometry (J Tech Commander PowerTrack II, UT, USA). Hand-held dynamometry has been demonstrated as a reliable procedure for assessing ankle strength [29]. The great toe was tested in isolation and the lesser toes assessed together. A ‘make’ test was used with force progressively increased to maximum level. Three attempts were used for each muscle group and the best attempt used in the analysis. Plantarflexor strength endurance was assessed using a standing single leg heel raise test [30]. Participants performed as many full single leg heel raises until fatigue. More detailed descriptions of the strength and endurance procedures used in the study have previously been published [31].

#### Joint flexibility

Ankle dorsiflexion range of motion was assessed using both the flexed-knee [32] and extended-knee lunge [33]. A digital inclinometer (Chattanooga Baseline, DJO global, CA, USA), placed on the anterior shank, was used to measure the angle of dorsiflexion. First metatarsophalangeal joint extension was measured in non-weight-bearing with a goniometer [34]. The ankle was positioned in plantargrade, with the first metatarsal head in line with the heel in the transverse plane. The hallux was dorsiflexed maximally by the tester, while preventing plantarflexion of the first ray and the angle formed by the lines connecting the navicular, the metatarsophalangeal joint axis and the middle of the interphalangeal joint was measured. As this procedure varied slightly from published reliability studies, pilot data were collected from 10 participants in order to determine intra-tester reliability. Measurements of first metatarsophalangeal joint extension were performed on each participant one week apart. Intra-class correlation coefficient was calculated to be 0.919, suggesting very high intra-tester reliability for this procedure.

A new technique to measure ankle inversion and eversion was conducted in side-lying using a digital inclinometer. For inversion, the participant was placed in side-lying with the leg being tested on the underside and the foot clear of obstructions. With the ankle in mid-range of plantarflexion and dorsiflexion, the foot was inverted fully. The inclinometer was zeroed on the shank just proximal to the malleolus, and then placed along the medial border of the calcaneus to obtain the inversion measure. With the foot placed into full eversion prior to zeroing the inclinometer, the same procedure was used to measure eversion. As this was a newly developed measurement procedure, pilot data were collected from 16 participants in order to determine intra-tester reliability. Measurements were performed on each participant one week apart. Intra-class correlation coefficients were 0.743 and 0.763 for the inversion and eversion range of motion measurements respectively, suggesting moderate to high intra-tester reliability.

#### Pain severity

Participants in the heel pain group assessed their pain level using a visual analogue scale (VAS). On a 100 mm line, with 0 representing no pain and 100 representing the most pain imaginable, participants were asked to mark the point that represented their heel pain at its worst [35]. Pain was also measured using the FHSQ pain domain [26]. This domain consists of four separate questions, answered on a Likert scale, focusing on the type, frequency and level of pain. Specific software (FHSQ version 1.03) converts the responses of each question into a total score between 0 and 100. A score of 100 represents no foot pain and a score of 0 represents the most severe experience of foot pain.

#### Pedobarography

Plantar pressure during barefoot walking was measured using the Emed® AT platform (Novel Gmhb, Munich, Germany). The platform features a sensor area of 360x190 cm housing 1377 sensors, a resolution of 2 sensors/cm^2^ and operates at a sampling rate of 25 Hz. Pressure data were captured using the reliable two-step method [36], with the participants walking at their normal, self-selected speed. Three successful trials were recorded, with trials discarded if part of the foot landed outside the sensor area; the participant targeted the platform, or reported that the step was not consistent with their normal gait. The average of the three attempts was used for each of these variables in each region of the mask heel divisions.

**Fig. 1:**
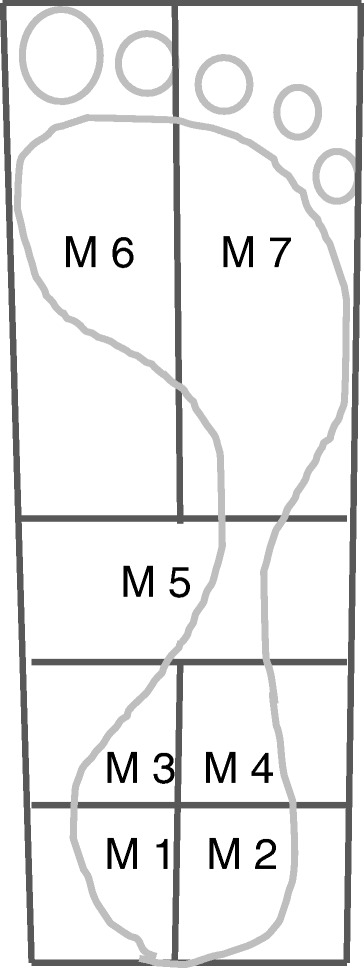
Mask generated using the Creation of Percentage Masks software (Novel, Gmbh, Germany). Rearfoot, midfoot and forefoot sections were created via divisions at 31 % and 50 % of the total foot length. The rearfoot section was subdivided into 4 equal quadrants through sagittal and frontal bisection, creating postero-medial, postero-lateral, antero-medial and antero-lateral heel regions. The forefoot section was bisected sagittally to create a medial forefoot and a lateral forefoot section

Pressure data were processed using using the Novel-win software package, version 8.07. (Novel Gmbh, Munich Germany). A mask [Fig. 1] was developed using the Creation of Percentage Masks software (Novel Gmbh, Munich Germany), enabling the foot to be divided into 3 sections: heel, midfoot and forefoot. These regions represent anatomically relevant regions of the foot based on skeletal measurements that allow clear and repeatable divisions of all feet [37]. The heel section measured the first 31 % of the total length of the foot, the midfoot section the next 19 % and the forefoot section the distal 50 %. The heel was sub-divided into four equal quadrants via sagittal and frontal plane bisection. The forefoot was bisected sagittally to create lateral and medial forefoot regions. Contact time, peak pressure, pressure–time integral, maximum force and force-time integral were collected for each region of the foot.

#### Data analysis

Descriptive statistics were computed and normality of data distribution determined with SPSS v22.0 (IBM SPSS Statistics for Windows, Armonk, NY, USA). The plantar heel pain groups were first compared for any differences in age and BMI using independent groups t-tests. Changes in foot morphology have been demonstrated with increasing age [38], with such changes affecting shoe fit in older people [39]. BMI has also been associated with alterations in foot morphology [40] and may potentially affect shoe fit. As such, any differences in age or BMI between groups would need to be controlled for in the subsequent analysis. A univariate analysis of covariance, with both age and BMI as covariates, was then constructed to compare the FHSQ footwear domain scores of the heel pain group with the control group to assess the impact of plantar heel pain on footwear comfort, fit and choice. Significance level was set at α = 0.05. Following this, the heel pain and control groups were compared on each of the three individual items of the FHSQ footwear domain, with the aim of highlighting which of footwear comfort, fit and choice were affected by the presence of heel pain. Mann Whitney U tests were used for this analysis as the data were Likert scale scores and not normally distributed.

Pearson’s correlation coefficients were calculated from the sample of participants with heel pain in order to explore which measures were associated with the FHSQ footwear domain score. Eleven variables significantly correlated with FHSQ footwear domain score (p < 0.05) were entered into the model sequentially to identify the best determinants of footwear-related difficulties. Only the most strongly associated variables were entered into the model, and to reduce multicolinearity, only one variable from highly correlated (r > 0.7) variables was used. In particular, foot dorsiflexion strength was used as a representative of overall ankle strength, lesser toe strength was used as the toe strength measure and VAS was used as the measure of foot pain.

## Results

The descriptive data for both groups are displayed in Table 1. When compared to the control group, people with plantar heel pain reported significantly poorer FHSQ footwear domain scores (mean difference −24.4; p < 0.001; 95 % CI: −32.0 to −17.0). Comparison between the heel pain and control groups on the three individual items of the FHSQ footwear domain score revealed reduced scores in the heel pain group for each one: comfort (p < 0.001), fit (p < 0.001) and number of shoes (p < 0.001).

**Table 1:** Participant characteristics of the sample. Data are mean and standard deviation (unless otherwise stated)

Characteristic	Plantar heel pain (n = 192)	Control (n = 69)	Significance
Age (years)	55.1 (13.3)	48.2 (17.1)	p = 0.001
Sex (% female)	68 %	61 %	p = 0.304
Weight (kg)	79.7 (16.5)	71.8 (14.2)	p < 0.001
Height (cm)	166.1 (8.8)	166.9 (9.7)	p = 0.516
BMI (kg/m^2^)	28.8 (5.2)	25.6 (3.8)	P < 0.001
Pain duration (months)	10 (4 – 24)^a^	0	-
Footwear score (FHSQ)	44.4 (27.0)	68.8 (28.0)	p < 0.001

^a^Median (inter-quartile range)

From the correlation matrix, the following variables were immediately discarded from the analysis due to non-significant correlation with the FHSQ footwear domain score: height, weight, BMI, symptom duration, Foot Posture Index, dorsiflexion range of motion (flexed knee and extended knee), first metatarsophalangeal extension range of motion, inversion and eversion range of motion, peak pressure in all regions of the foot, pressure–time integrals in the heel, midfoot and lateral forefoot, maximum force under the forefoot and the medial heel and force-time integral at the forefoot. Eleven variables were retained for the multiple regression analysis and further variables then removed if their association with the FHSQ footwear domain score did not reach statistical significance (p < 0.05). These comprised age, pain level, ankle strength, calf endurance, maximum force at the antero-lateral heel, force-time integrals at the medial heel, pressure–time integral in the medial forefoot, and foot contact time during walking. The final regression model is detailed in Table 2. Three variables were retained in the regression model, explaining a total of 15 % of the variance in the FHSQ footwear domain score (r^2^ = 0.150). These variables included: maximum force under the postero-lateral heel when walking barefoot, toe flexor strength and gender. Specifically, a lower FHSQ footwear domain score, representing greater difficulty with footwear, was associated with reduced maximum force under the postero-lateral heel when walking barefoot, toe flexor weakness and female gender. Toe flexor strength and VAS heel pain score did not correlate significantly (r = −0.083; p = 0.245).

**Table 2:** Regression model: variables associated with FHSQ footwear domain score (n = 192)

Variable	B	SE	Beta	p value	R^2^
Toe strength	.214	.070	.220	.003	
Heel force	.950	.329	.201	.004	.150
Gender	−8.172	4.071	-.142	.046	

## Discussion

There are two main findings from this study. First, people with plantar heel pain have significantly greater difficulty with footwear comfort, fit and choice compared to unaffected individuals, which is in agreement with previous research [13]. Second, among those with heel pain, reduced heel loading during walking, lower toe flexor strength and female gender are associated with greater footwear difficulties. A number of key variables measured were removed from the regression model and appear to have no association with the FHSQ footwear domain score. These include: pain level, age, BMI, foot and ankle range of motion, ankle strength, calf endurance, foot contact time during walking and foot posture.

The lower mean FHSQ footwear domain score of the heel pain group suggests that simply having plantar heel pain produces difficulty with footwear. Furthermore, people with plantar heel pain appear to have difficulty with all three components of the footwear domain, namely; comfort, shoe fit and number of shoes able to be worn. It is likely people with heel pain have difficulty finding shoes that can accommodate weight-bearing stresses sufficient to protect against pain, and this could include difficulty finding one or multiple shoes capable of minimising heel pain. Shoes are made from a variety of materials with different mechanical properties, suggesting people with heel pain may find particular shoes painful to walk in, but not necessarily all. Reported difficulties with shoe fit related to heel pain was a less expected finding. High level evidence linking foot posture to plantar heel pain is lacking and it has not been found to be associated with any particular foot morphology or deformity [25]. In this study, foot posture was not found to be related to footwear difficulties; however, other measures of foot morphology such as width, length and height were not included. Future studies are needed to investigate morphological characteristics of the foot in an attempt to better understand issues of shoe fit in relation to plantar heel pain. It is possible that people who experience difficulties with shoe fit have their foot function affected by poorly fitting shoes, with this predisposing them to heel pain. Alternatively, people with heel pain commonly experiment with various shoe inserts which could potentially affect footwear fit. While orthoses have been shown to be effective as an intervention for plantar heel pain [25], future studies are needed to assess whether they have an adverse effect on footwear fit.

Maximum force beneath the postero-lateral heel was the loading parameter that correlated most with FHSQ footwear score and was retained in the final regression model. A reduction in maximum loading of this region suggests a strategy to reduce initial impact at the heel during walking. People with heel pain who adopt this strategy appear to have more difficulty with footwear selection, possibly because they would require shoes that are very effective in absorbing impact at the heel. Given the influence of pain presence and heel loading on footwear accessibility, footwear-based interventions seem likely to play an important role in the management of heel pain. Different types of footwear have been demonstrated to alter pressure distribution and pain levels in foot conditions such as gout [41, 42] and rheumatoid arthritis [43]. One study has reported reduction in pain and reduced heel pressures using rocker-soled footwear in people with heel pain, with greater effect evident if used in combination with custom orthoses [23]. Further research is required to compare pain levels and load distribution using different types of footwear in people with heel pain. In particular, studies using in-shoe pressure measurement could evaluate which footwear provides the most effective load reduction beneath the heel and greatest decreases in pain, as this would assist clinicians with dispensing footwear advice to people with heel pain.

Toe flexor strength was retained as a predictor variable in the regression model, suggesting a link between toe flexor weakness and greater footwear-related difficulties in people with heel pain. This appears to be a relationship that is independent of pain severity, as toe flexor strength and VAS heel pain score did not correlate significantly. It may be that difficulty accessing footwear indirectly affects muscle function by reducing activity levels in people with heel pain. People who have difficulty finding comfortable footwear may become limited regarding what they are able to do physically. Such reduction in physical activity, in turn, could lead to reduced muscle strength. Longitudinal studies would be needed to distinguish whether toe weakness occurred subsequent to footwear selection difficulties and reduced activity. If this were the case, it would further emphasise a need for effective footwear advice and interventions for people with heel pain to enhance activity and reduce possible impairments.

Gender was retained in the regression model suggesting that females with plantar heel pain are more likely than males to have difficulties with footwear comfort, fit and choice. Women are far more likely than men to wear shoes considered to be poor in terms of structure, support and risk [44]. Women commonly wear ill-fitting shoes that are narrower relative to their foot size, relating to various types of foot pain and deformity [45, 46]. Some evidence exists for aesthetics being the most influential factor in footwear selection for older women [47], with this potentially contributing to comfort levels of shoes chosen. In addition, high-heeled shoes worn by women have been shown to modify foot and lower limb kinematics and kinetics, potentially contributing to symptoms [48]. This suggests that, while the presence of heel pain is associated with footwear difficulties, added issues with footwear comfort, fit and choice are apparent in females who have the condition.

Importantly, whilst three variables were identified as being associated with footwear-related difficulties in people with heel pain, the regression model was only able to explain 15 % of the variance of the FHSQ footwear domain score. This suggests that factors other than those included in this study must represent a considerable amount of the variation in self-reported footwear comfort, fit and choice.

Limitations of the study include the cross-sectional design, meaning that conclusions cannot be made regarding cause-and-effect when considering associations between individual variables and FHSQ footwear domain score. Plantar pressure measures were not collected in-shoe and were, therefore, not reflective of foot loading whilst shod. However, plantar pressure data collected barefoot standardises testing across the sample and is indicative of an individual’s willingness to load the foot. Lastly, a single examiner collected all of the data and was not blind to group membership, leaving examiner bias unable to be discounted entirely. Despite these limitations, our findings have identified factors associated with footwear difficulties in people with heel pain which may have important implications regarding management of the condition.

## Conclusion

People with plantar heel pain report difficulty with footwear comfort, fit and choice. Factors associated with footwear difficulties in people with heel pain include a reduced heel loading strategy during walking, weaker toe flexors and female gender. Future research is needed to investigate the effect of different footwear to reduce plantar heel pain, as well as issues relating to shoe fit, with the aim of optimising footwear recommendations as part of a comprehensive management approach.
